# Supplementary material for: Epicatechin-Loaded Nanocapsules: Development, Physicochemical Characterization, and NLRP3 Inflammasome-Targeting Anti-Inflammatory Activity
Source: Biology (Basel). 2025 Oct 30;14(11):1520. doi: 10.3390/biology14111520 (PMC12649874; doi:10.3390/biology14111520)

Figure S1: Average size (A) and zeta potential (B) graphs of NC-EC.

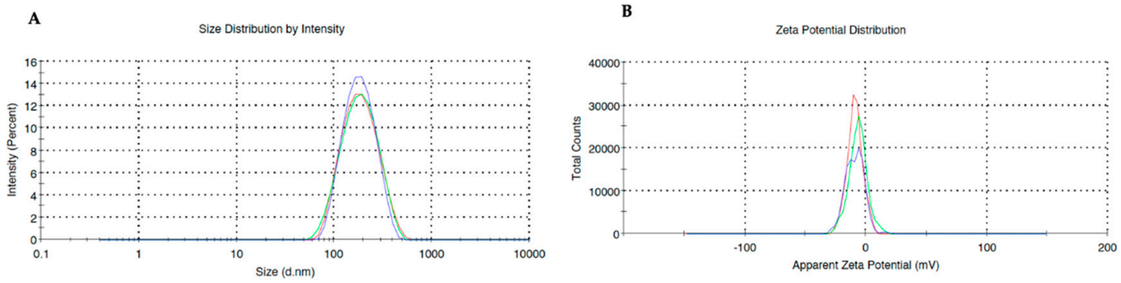

Figure S2: Average size (A) and zeta potential (B) graphs of NC-B.

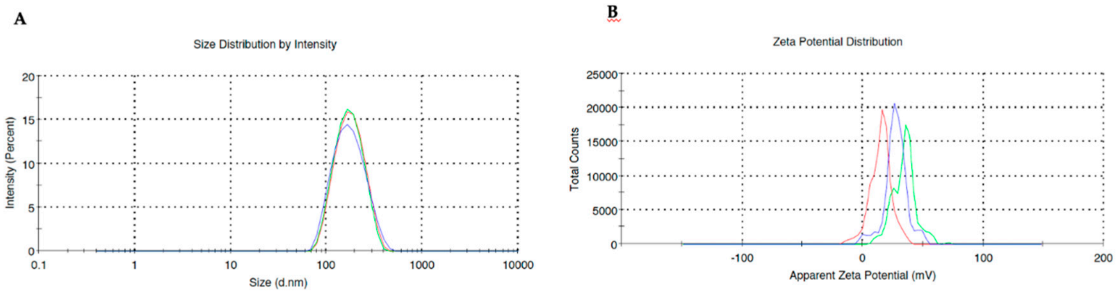

Supplementary Material Table S1 – Thermal stability analysis of epicatechin-loaded nanocapsules (NC-EC) [0.25 mg/mL].

Sample	Time (days)	Climate condition	Size (nm) ± SD	PDI ± DP	Zeta potential (mV) ± SD	pH ± SD
NC-B	0	-	177,0 ± 1,65	0,222 ± 0,01	-11,46 ± 0,90	3,80 ± 0,02
	7	RE	172,5 ± 9,40	0,182 ± 0,04	-14,4 ± 1,05	4,05 ± 0,04
	15	RE	169,8 ± 5,9	0,178 ± 0,02	-10,2 ± 1,93	3,95 ± 0,08
	30	RE	166,2 ± 2,04	0,160 ± 0,01	-11,2 ± 2,22	4,26 ± 0,01
	45	RE	168,3 ± 2,90	0,132 ± 0,002	-8,71 ± 0,43	4,22 ± 0,01
	60	RE				
	90	RE				
NC-EC$_1$ [0,25 mg/mL]	0	-	156,1 ± 0,92	0,111 ± 0,01	-11,36 ± 1,24	3,85 ± 0,02
	7	RT	160,5 ± 0,83	0,126 ± 0,01	-17,8 ± 0,51	3,98 ± 0,06
		RE	174,3 ± 4,42	0,219 ± 0,01	-9,68 ± 1,31	4,09 ± 0,09
		CC	161,9 ± 1,05	0,144 ± 0,01	-10,45 ± 0,95	4,26 ± 0,03
	15	RT	162,9 ± 0,30	0,127 ± 0,02	-13,3 ± 0,83	4,17 ± 0,005
		RE	162,5 ± 2,01	0,164 ± 0,007	-10,4 ± 0,37	4,04 ± 0,04
		CC	194,8 ± 5,02	0,23 ± 0,023	-9,6 ± 3,39	4,23 ± 0,02
	30	RT	159,1 ±2,11	0,134 ± 0,01	-18,7 ± 1,4	4,35 ± 0,005
		RE	155,4 ± 1,15	0,126 ± 0,01	-15,1 ± 1,30	4,23 ± 0,03

		CC	162,3 ± 2,37	0,094 ± 0,007	-21,6 ± 1,92	4,54 ± 0,00
	45	RT	205,3 ± 1,45	0,244 ± 0,006	-23,2 ± 2,31	4,39 ± 0,02
		RE	160,6 ± 2,96	0,090 ± 0,02	-8,49 ± 0,71	4,27 ± 0,03
		CC	178,6 ± 9,29	0,152 ± 0,041	19,3 ± 0,88	4,5 ± 0,02
	60	RT				
		RE				
		CC				
	90	RT				
		RE				
		CC				
NC-EC2 [0,25 mg/mL]	0	-	156,4 ± 0,75	0,134 ± 0,20	-8,37 ± 0,90	3,85 ± 0,02
	7	RT	169,9 ± 0,73	0,139 ± 0,01	-18,2 ± 1,70	3,70 ± 0,01
		RE	156,13 ± 0,70	0,113 ± 0,005	-14,1 ± 0,81	3,91 ± 0,02
		CC	159,0 ± 1,80	0,144 ± 0,001	-15,3 ± 1,65	3,99 ± 0,005
	15	RT	181,3 ± 0,70	0,137 ± 0,01	-18,2 ± 1,62	3,74 ± 0,05
		RE	162,3 ± 2,46	0,165 ± 0,03	-13,8 ± 1,19	3,92 ± 0,05
		CC	159,2 ± 1,36	0,150 ± 0,01	-15,3 ± 0,61	3,90 ± 0,01
	30	RT	206,1 ± 13,9	0,250 ± 0,04	-19,7 ± 1,13	4,01 ± 0,00
		RE	159,4 ± 0,69	0,152 ± 0,01	-11,2 ± 0,91	4,19 ± 0,02
		CC	163,7 ± 5,68	0,190 ± 0,02	-8,47 ± 2,22	4,21 ± 0,02
	45	RT	193,3 ± 3,68	0,174 ± 0,026	-23,16 ± 0,85	4,07 ± 0,01
		RE	161,3 ± 4,12	0,155 ± 0,02	-10,73 ± 0,37	4,15 ± 0,02
		CC	162,4 ± 2,02	0,157 ± 0,01	-21,76 ± 1,30	4,09 ± 0
	60	RT				
		RE				
		CC				
	90	RT				
		RE				
		CC				
NC-EC3 [0,25 mg/mL]	0	-	154,2 ± 1,53	0,118 ± 0,01	-13,5 ± 0,75	3,85 ± 0,02
	7	RT	197,7 ± 0,87	0,184 ± 0,01	-19,1 ± 1,30	3,58 ± 0,05
		RE	175,3 ± 1,32	0,150 ± 0,005	-17,9 ± 0,72	3,84 ± 0,005
		CC	188,2 ± 5,69	0,202 ± 0,01	-16,5 ± 1,36	3,94 ± 0,01

	15	RT	181,8 ± 0,70	0,137 ± 0,01	-18,2 ± 1,62	3,68 ± 0,03
		RE	162,3 ± 2,46	0,165 ± 0,03	-13,8 ± 1,19	3,88 ± 0,05
		CC	181,9 ± 2,95	0,181 ± 0,009	-14,7 ± 0,79	3,89 ± 0,06
	30	RT	208,9 ± 14,6	0,197 ± 0,04	-12,2 ± 1,91	3,92 ± 0,01
		RE	178,4 ± 6,9	0,172 ± 0,03	-16,7 ± 1,21	4,19 ± 0,005
		CC	194,5 ± 4,20	0,208 ± 0,01	-16,8 ± 1,53	4,15 ± 0,005
	45	RT	204,6 ± 1,58	0,168 ± 0,03	-28,2 ± 2,08	4,06 ± 0,03
		RE	176,8 ± 4,78	0,162 ± 0,02	-19,3 ± 2,23	4,24 ± 0,03
		CC	187,0 ± 2,75	0,166 ± 0,01	-21,4 ± 1,88	4,20 ± 0,005
	60	RT				
		RE				
		CC				
	90	RT				
		RE				
		CC				

Legend: [1] Lot 01; [2] Lot 02; [3] Lot 03.

Note: After 60 days of analysis, samples from all batches and storage temperatures presented a foul odor and the formation of microorganism colonies on the bottom and sides of the vials. Therefore, the analysis was discontinued, with stability remaining for up to 45 days, and refrigeration being the best storage condition.

Supplementary Material Figure S3 – NC-EC *in vitro* safety profile.

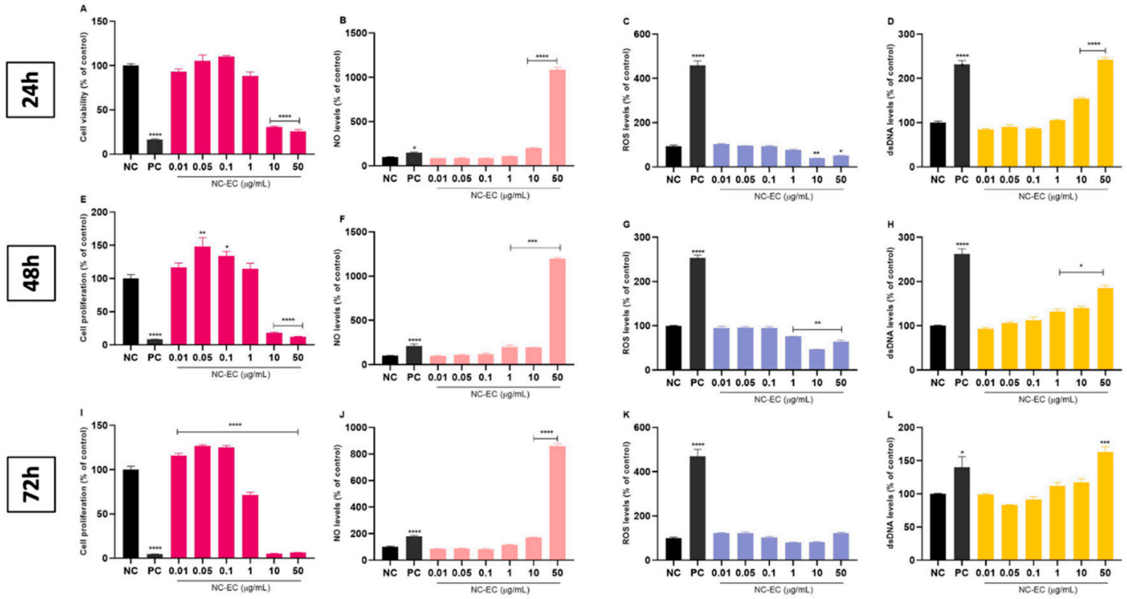

Legend: NC-EC concentration curve - *In vitro* safety profile evaluation. VERO cells were exposed to different concentrations of NC-EC for 24, 48, and 72 h of incubation. **(a, e, and i)** Evaluation of predictive (24 h) and cellular regularity (48 and 72 h) indices by the MTT assay; **(b, f, and j)** Measurement of NO levels after 24, 48, and 72 h of incubation, respectively; **(c, g, and k)** Measurement of ROS levels after 24, 48, and 72 h of incubation, respectively; **(d, h, and l)** Quantification of extracellular dsDNA indices after 24, 48, and 72 h of incubation, respectively; NC: negative control (untreated cells); CP: cells exposed to 200 µM H2O2 for MTT, DCFH-DA and PicoGreen assays and 10 µM sodium nitroprusside for NO determination assay; Statistical analysis was performed by one-way ANOVA followed by Tukey's post hoc test. Results with p<0.05 were considered significant. *p<0.05; **p<0.01; ***p<0.001; ****p<0.0001.

Supplementary Material Figure S4 – NC-B *in vitro* safety profile.

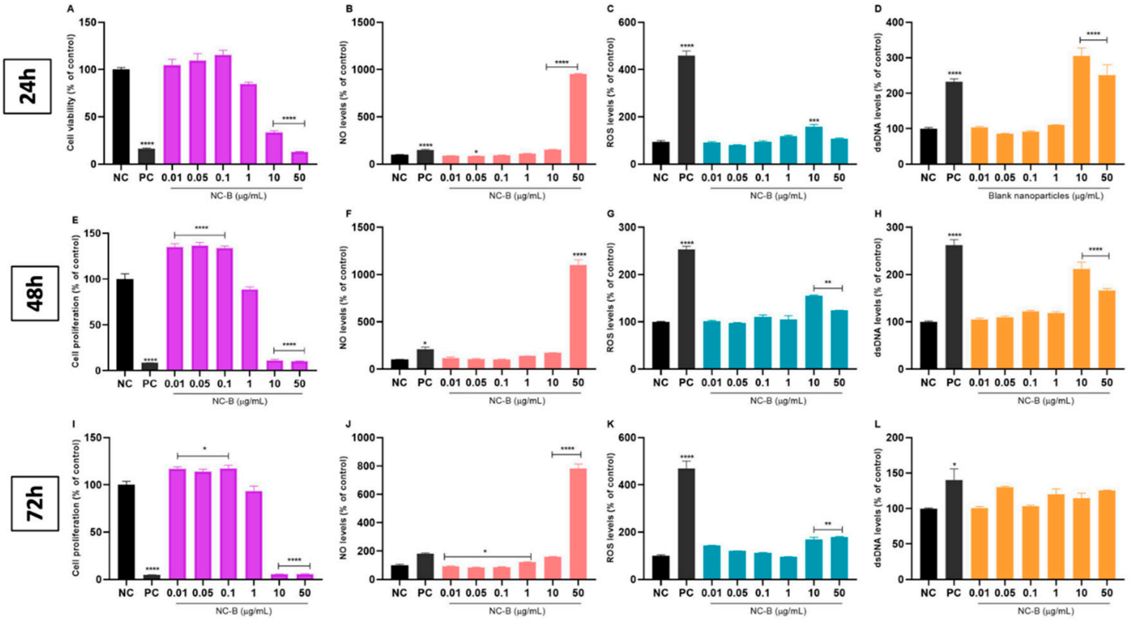

Legend: NC-B concentration curve - *In vitro* safety profile evaluation. VERO cells were exposed to different concentrations of NC-EC for 24, 48, and 72 h of incubation. **(a, e, and i)** Evaluation of predictive (24 h) and cellular regularity (48 and 72 h) indices by the MTT assay; **(b, f, and j)** Measurement of NO levels after 24, 48, and 72 h of incubation, respectively; **(c, g, and k)** Measurement of ROS levels after 24, 48, and 72 h of incubation, respectively; **(d, h, and l)** Quantification of extracellular dsDNA indices after 24, 48, and 72 h of incubation, respectively; NC: negative control (untreated cells); CP: cells exposed to 200 µM H2O2 for MTT, DCFH-DA and PicoGreen assays and 10 µM sodium nitroprusside for NO determination assay; Statistical analysis was performed by one-way ANOVA followed by Tukey's post hoc test. Results with p<0.05 were considered significant. *p<0.05; **p<0.01; ***p<0.001; ****p<0.0001.

Supplementary Material S1 – Ultrafiltration validation.

The statistical analysis (unpaired t-test, $p = 0.5258$) indicated no significant difference between the concentrations of the bioactive compound obtained for the theoretical standard and the standard after the ultrafiltration and centrifugation processes. This result demonstrates that the total amount of active compound recovered after the procedure was equivalent to that initially present in the standard sample, indicating that ultrafiltration did not lead to retention of the bioactive compound in the filter. These findings support the reliability of the previously reported encapsulation efficiency results for epicatechin in the nanostructures.

(a) Standard epicatechin content.

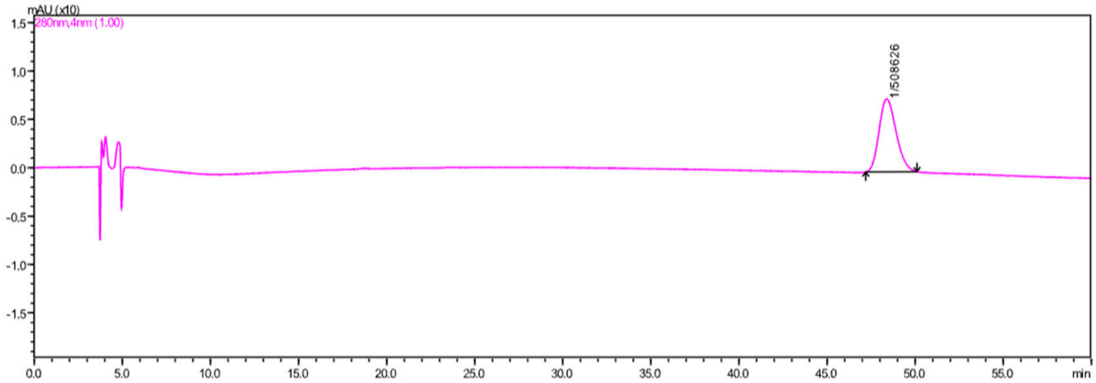

(b) Device standard test for encapsulation efficiency

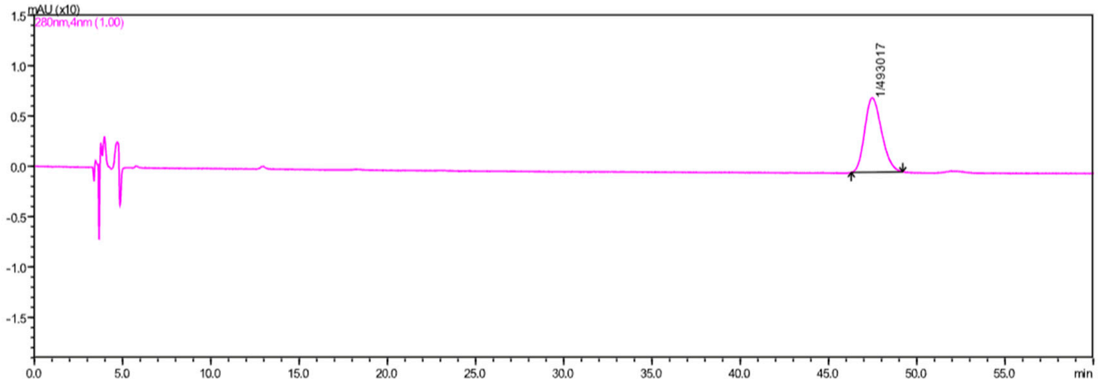

Supplement: Supplementary file 1 [file biology-14-01520-s001.zip › biology-3892344-supplementary.pdf]
